# Socio-demographic correlates and trends in the timing of the onset of parenthood among women of reproductive age in Ghana: evidence from three waves of the demographic and health surveys

**DOI:** 10.12688/f1000research.130349.1

**Published:** 2023-02-10

**Authors:** Ololade Julius Baruwa, Yaw Acheampong Amoateng

**Affiliations:** 1 School of Public Health, University of the Western Cape, Cape Town, South Africa; 2 Population and Health Research Entity, Faculty of Humanities, North-West University, Mafikeng, North West Province, 2735, South Africa

**Keywords:** onset of parenthood, sociodemographic, childbearing, Ghana, Demographic and Health Surveys, Cox proportional hazard Women

## Abstract

Background: Childbearing is one of the central events in a woman’s life and the age at which this event occurs has important health, socioeconomic and fertility implications for her.

Methods: We used three waves of the Ghana Demographic and Health Surveys (GDHS) from the individual files of married women aged 15 to 49 years old to explore the trends in the timing of the onset of parenthood among women in Ghana. The Cox proportional hazard model was used to assess the effect of socio-demographic factors on the birth experience of women.

Results: Results showed the median age of the women increases from 17 years in 1998 to 19 years in 2014. Further, results showed that women with secondary education had 0.67, 0.89- and 0.77-times lower hazard risk of early birth than women without any formal education in 1988, 1998, and 2014 respectively. The hazard risk of early childbirth consistently decreased as age increased in all the years of surveys except in the case of the age group 40-44 in 1988, 1998 and 2014.

Conclusions: This study showed that the timing of first childbirth is changing in the direction of a late childbirth regime in Ghana and could facilitate improvement on individual health, job stability and higher level of education. Efforts should be channeled to sensitizing women on the importance of delaying childbearing.

## Introduction

Childbearing is one of the central events in a woman’s life course and the age at which this event occurs has important health, socioeconomic and fertility implications for her. For instance, the age at which a woman has her first birth is a risk factor for poor maternal health throughout the life course ( Schiücker and Blumenfelder 2014; Blanc, Winfrey, and Ross 2013). Childbearing at adolescence may also lead to physiological dysfunctions that may lead to chronic diseases and physical disability in later life ( Cho *et al.* 2012). In settings where resources are limited, early age at first birth has been associated with higher maternal mortality ( Gupta and Mahy 2003) and has adverse effects on child health and child survival because of low birth weight and pre-term births which lead to infant mortality ( Wendt *et al.* 2012; Gibbs *et al.* 2012). Other reported effects of early age at first birth are truncated education which has ramifications for the socioeconomic wellbeing of women and their children ( Akella and Jordan 2015; Leung, Groes, and Santaeulalia-Llopis 2016; Gibbs *et al.* 2012; Jutte *et al.* 2010).

Because of the above-mentioned effects and implications of the timing of first birth, the existing literature is replete with studies examining the problem of how social, economic, and demographic conditions impact the way in which women have their first child in both developed and developing societies (e.g. Aksan 2014; Ayiga 2015; Bongaarts and Ann 2015; Gibbs *et al.* 2012; Karabo and Ayiga 2014; Rabbi and Kabir 2013; Statistics South Africa 2015; Tadesse 2014; Takyi and Addai 2002). In Ghana, there is strong evidence that age at first birth has a strong impact in explaining the fertility of contemporary cohorts. That is, the timing of the first birth is essential in determining the completed fertility among Ghanaian women ( Obeng Gyimah 2003). Recent evidence shows that fertility transition is notable in Ghana. For instance, the 1988, 1998 and 2014, Ghana Demographic and Health Survey (GDHS) statistics shows that the median age at first birth increased from 17 in 1988 to 19 in 2014. However, as the brief review of studies has clearly shown, studies that look at the socio-demographic trend in the timing of first birth are almost non-existent. Invariably, almost all the studies on timing of first birth have been examining the problem at a single point in time, ignoring the question of trend over time. Although a previous study in Ghana reported that more than half (51.4%) of women of reproductive age have given birth at the age of 19 ( Amoateng 1991) while another study revealed the median age at first birth in Ghana is 20 years ( Obeng Gyimah 2003). However, these studies are rather too old ( Amoateng 1991; Obeng Gyimah 2003). Besides, evidence linking the sociodemographic factors of women and timing of age at first birth over a period is lacking in Ghana.

It is against the backdrop of the foregoing effects of the timing of first birth that we undertake the present study. The aim of the present study is to examine the effect of selected socio-demographic factors on the age at which women have their first child in Ghana. The present study becomes important in addressing the void in the existing body of knowledge, the study goes beyond these existing studies by using three waves of the demographic and health survey data on Ghana to examine trend in age at first birth. Consistent with the study’s aim of examining change over time, we use a process-oriented theoretical framework-the family life course perspective to examine trends in the timing of first birth over three waves of the Ghana Demographic and Health Survey data.

### Theoretical framework and review of the empirical literature

The present study used the Life Course Theory, theorized by Glen Elder ( Elder, 1998). The Life Course Theory helps in understanding how the individual interfaces social institutions (e.g., education, economy, occupation, place of residence etc.) through rational choices as he/she goes through life. The theory directs attention to the powerful connection between individual lives and the historical and socioeconomic contexts in which these lives unfold. As a concept, a life course is defined as “a sequence of socially defined events and roles that the individual enacts over time” ( Giele and Elder 1998). Since a household is basically a confluence of people at different points in their life courses, age becomes a crucial factor in the analysis of family change because the cumulative experiences of these individuals help to define the family as a social institution ( De Vos 1995).

The life course perspective emphasizes the importance of time, context, process, and meaning on human development and family life ( Bengtson and Katherine 2009), while the family is perceived as a micro social group within a macro social context. The life course reflects the intersection of social and historical factors with personal biography and development within which the study of family life and social change can ensue ( Elder 1998).

In most developing countries, especially in sub-Saharan Africa, where early childbirth is common, there has been visible transition in this pattern of family building as a result of modernizing influences such as urbanization and education ( Gibbs *et al.* 2012), and improvements in child health and survival ( Aksan 2014). The existing literature is replete with studies that have identified the effects of education and urbanization on the age at which women have their first child. For example, a study found that lack of education or low education does not only lead to early marriage, but it also impedes access to contraception leading to unintended pregnancy and thus early age at first birth for women ( Bongaarts and Ann 2015). On the other hand, better educated women initiate childbearing at older ages, mainly because education delays age at first birth since being in school is not compatible with childbearing ( Karabo and Ayiga 2014). Moreover, education increases age at first birth through engendering favorable attitudes towards and uptake of contraception.

Place of residence has also been found to be a predictor of age at first birth through its indirect influence on the age at first union (marriage or cohabitation) and ease of access to contraceptive use by sexually active women in urban areas. Existing studies in sub-Saharan Africa suggest that the age at marriage is early in rural areas because the return to education, an important factor that increases age at birth is low in rural areas ( Tadesse 2014), while the relative lack of access to contraceptives in rural areas increases the risk of early pregnancy and childbearing ( Ayiga 2015). In South Asia, where the age at first birth is early (less than 18 years), Rabbi and Kabir (2013) observed that it was also attributable to the early age at marriage and poor access to contraceptives in rural areas.

Besides education and urban living, life course events such as changes in the age at marriage have been observed worldwide as one of the important proximate determinants of age at first birth ( Gupta and Mahy 2003). For example, the generally early age at first marriage regime in sub-Saharan Africa has been found to be a factor influencing the high fertility rates in these countries since childbearing begins soon after marriage and continues for a long time thereafter ( Khan *et al.* 2008). Conversely, if marriage occurs at an older age, the age at first birth will also be much higher ( Chung *et al.* 2006).

Age at first birth has been found to be affected by socio-cultural factors such as religion, ethnicity and region of residence. As far as religion is concerned, some studies have found that women who subscribe to a more pro-natalist religious doctrine, i.e. religions that reinforce family values and prohibit contraception or abortion such as Catholicism, are more likely to have earlier first births ( McQuillan 2004). However, in a study of Ugandan women aged 15-49 years old, Mugarura, Kaberuka and Atuhaire (2016) found religion to be an important determinant of age at first birth. They found that Muslim women were 1.67 times more likely to give birth at an early age compared to Catholic women, Pentecostal women 1.49 times more likely to have an early birth compared to Catholic women, and Seventh Day Adventist (SDA) women 1.8 times more likely to have a child at an early age compared to Catholic women.

In Tanzania, Ngalinda (1998), found that Muslim women were more likely to get their first child earlier than any other religious denomination in Tanzania ( Ngalinda 1998). However, unlike many previous studies, Ngalinda (1998) found Catholic women in Tanzania to have a higher mean age at first birth (18.6 years) compared to Protestants (18.5 years). However, this is contrary to findings by Logubayom & Luguterah (2015) who found that religious affiliation was not a significant determinant of Ghanaian women’s waiting time to first birth. In terms of region, the Ghana Statistical Service (2009) found that the median age at first birth across regions showed earlier first births for women in Northern Ghana (i.e., 19.5 years in the Upper East and Upper West regions) compared to their Southern counterparts (e.g. 23.2 years in Greater Accra).

Ethnicity which reflects an individual’s cultural background has been found to be associated with reproductive decisions or family formation, although its effects on age at first birth has not received much attention in the literature. In Nepal, ethnicity was found to have large impact on early motherhood ( Choe, Thapa & Mishra 2005), while in Ghana, Takyi and Addai (2002), using data from the early 1990s, did not find ethnicity to be an important predictor of family formation in Ghana. Using Census 2011 data and language as a proxy for ethnicity in South Africa, it was found that Xhosa, Zulu, SiSwati, Tshivenda and Tsonga speaking women aged 45–49 had a lower median age at first birth compared to English speaking and Afrikaans speaking women ( Statistics South Africa 2015).

### Objective of the study

To the best of our knowledge of the existing literature, there are no studies conducted on the socio-demographic factors of the timing of age at first birth in Ghana. However, the availability of several DHS in Ghana conducted between 1988 and 2014 provides us with an opportunity to use these data sets to examine trends in age at first birth in Ghana. Therefore, the aim of the present study is to examine the effect of selected socio-demographic factors on the age at which women have their first child in Ghana.

## Methods

### Data and sample

The present study employs data from the Ghana Demographic and Health Surveys (GDHS) from the women individual files of ever married women aged 15 to 49 years old for the survey periods 1988, 1998 and 2014 (4481, 4843 and 9396 respectively). There is no methodological or substantive reason why the present analysis is limited to only three out of the six waves of the survey other than the fact that we try to look at the trends that have occurred with an interval of at least 10 years. Ten years is the benchmark the United Nations has set in the decennial census programme as it is considered long enough to measure changes in both the independent and outcome variables.

Data collected across the three surveys included background characteristics such as education, ethnicity, religion, place of residence, region of residence, age at first sexual intercourse, age at first marriage or cohabitation, age at first birth, marital duration, total number of children ever born, health service providers, communities, household health expenditures of women as well as information on young adults.

The outcome variable for this study is onset of parenthood (age at first birth), while the explanatory variables of interest are variables that have been found to be associated with age at first birth. These explanatory variables include age (measured as 15-19, 20-24, 25-29. 30-34, 35-39, 40-44, and 45-49), place of residence (measured as rural and urban), ethnic group (measured as Akan, Ga-adangbe, Ewe, Guan, Mole-dagbani, and other), education (measured as no education, primary, secondary, and tertiary), marital status (measured as never married and ever married), religion (measured as no religion, Catholic, other Christian, Muslim and traditional), region (measured as western, central, greater/Accra, Eastern, Volta, Ashanti, B/A and northern.

### Sampling design

The GDHS used a two-stage sample design and was intended to provide estimates of key indicators at the national, urban, and rural levels, as well as for each of Ghana’s ten administrative regions. The first step was to select sample points (clusters) made up of enumeration areas (EAs). The second stage involved systematic household sampling. Even though there were slight variations in the sampling designs over the years, invariably, all the surveys used clusters from which representative samples of women were selected. The EAs consisted of three strata, namely, Coastal Savannah, Forest and Northern Savannah, and within each stratum, urban, semi-urban and rural EAs were identified giving an average of about 400 EAs. The combined effective sample size for the three data sets is 18,727 women aged 15-49 years.

### Research tools and data collection

Three questionnaires were used in the GDHS: the Household Questionnaire, the Woman's Questionnaire, and the Man's Questionnaire. These questionnaires were adapted from standard Demographic and Health Survey (DHS) questionnaires to reflect the population and health issues relevant to Ghana. Various stakeholders representing government ministries and agencies, nongovernmental organizations, and international donors were asked to provide feedback on the questionnaires. The final questionnaires were written in English first, then translated into the major local languages of Akan, Ga, and Ewe.

The women’s questionnaire was used to gather information from all eligible women aged 15-49 years. These women were questioned on the following topics; background characteristics, birth history and child mortality, knowledge and use of family planning, fertility preference, marriage and sexual activities, antenatal and delivery among others. Staff members from the Ghana Statistics Services (GSS) and the Ghana Health Service (GHS) supervised and coordinated the fieldwork. Data collection took place at the respondents’ houses. For 1988 GDHS, data collection and fieldwork began in March and lasted until June 1988. For 1998 GDHS, data collection began in November 1998 and ended in February 1999. For the 2014 GDHS, data collection took place between August and December 2014. The GDHS are mostly self-reported, while the fieldworkers did the data entry.

### Statistical analyses

Before the commencement of statistical analyses, the data were weighted using the data sampling weight to account for the differentials in the sample design and response rate. A descriptive analysis was conducted using univariate analysis to describe the median age, which gives a better picture of age distribution at first birth and the background characteristics, while Cox proportional hazard model was employed for the multivariate analyses ( Cox 1972). In the DHS survey, age at first birth is given as the woman’s age (at her last birthday) at the time of her first birth.

The Cox proportional hazard model assesses the effect of socio-demographic factors on the birth experience of women in a multiple regression framework. Following Cox (1972), the Cox model is defined as:

$$h\left(t\right)=h_{0}\left(t\right)\times \exp\left\{b_{1}x_{1}+b_{2}x_{2}+\ldots +b_{p}x_{p}\right\}$$

Where the hazard function $h\left(t\right)$ is the dependent variable, which is dependent on a set of p covariates $\left(x_{1},x_{2},\ldots ,x_{p}\right)$ whose impact is measured by the size of the respective coefficients $\left(b_{1},b_{2},\ldots ,b_{p}\right)$ . The term $h_{0}$ is the baseline hazard, which gives the value of the hazard if all the $x_{i}$ are equal to zero. Empirically, estimating the Cox regression involves the status, time and covariate variables. The status variable is the dependent variable in the regression is a binary response, which is the birth experience of a woman. Therefore, the dependent variable is whether or not first birth has occurred. The responses are coded 0 for those women who indicate that they have never had a child, and 1 for those who have ever been a parent.

In the present analysis, we disaggregated the result for each survey by year of survey in order to observe the effects of the independent variables on the dependent variables over the period of each survey. The results are presented as hazard risk, showing for example, the relative likelihood of a woman in a given educational category having a first birth compared to a woman with tertiary education that is used as the reference category. A variable has a significant effect on age at first birth if the associated relationship is significant at p<0.05.

### Ethical considerations

This research utilized secondary information that was collected from 1988, 1998, and 2014 GDHS. Ethical authorization to utilize this information was granted by ICF Macro through the DHS programme site ( dhsprogram.org). The DHS fieldworkers were trained on how to obtain informed consent from the participants, and ensured that the participants knew they could withdraw from the survey at any time. Hence, the dataset used in this study doesn’t include identifying or other confidential information about the respondents. In this manner, obscurity and privacy of the study participants were ensured.

## Results

### Background characteristics

The socio-demographic characteristics of women aged 15-49 years are presented in Table 1. The table shows that the distribution of the women by five-year age intervals is a normal bell-shaped curve with a peak at the 20-24 and 25-29 age group in 1988 and thereafter declines monotonically to 45-49 years age group. However, for 1998 and 2014, the majority of the women are 15-19 years of age and the proportion of this age group declines linearly thereafter to age group 45-49. The distribution of women by place of residence shows that although the proportion of women in urban areas increased to 49% in 2014, the women remained predominantly rural residents in all the survey years.

**Table 1.** Socio-demographic characteristics of women aged 15-49 years in Ghana 1988, 1998 and 2014 DHS.

	1988 (4481)	1998 (4843)	2014 (9396)
**Variables**			
**Age**			
15-19	849 (18.9)	889 (18.4)	1756 (18.7)
20-24	867 (19.3)	887 (18.3)	1571 (16.7)
25-29	867 (19.3)	857 (17.7)	1564 (16.7)
30-34	644 (14.4)	661 (13.7)	1343 (14.3)
35-39	531 (11.8)	627 (12.9)	1260 (13.4)
40-44	364 (8.1)	484 (10.0)	1032 (11.0)
45-49	366 (8.1)	438 (9.0)	870 (9.3)
**Place of residence**			
Urban	1523 (33.9)	1585 (32.7)	4602 (49.0)
Rural	2965 (66.1)	3258 (67.3)	4794 (51.0)
**Level of education**			
No education	1783 (1783)	1737 (35.9)	2281 (24.3)
Primary	2369 (52.8)	813 (16.8)	1747 (18.6)
Secondary	296 (6.6)	2188 (45.2)	4854 (51.7)
Tertiary	40 (0.9)	105 (2.2)	514 (5.5)
**Marital status**			
Never married	889 (19.8)	1092 (22.6)	3041 (32.4)
Ever married	3598 (80.2)	3751 (77.5)	6355 (67.4)
**Ethnic groups**			
Akan	2379 (53.1)	2240 (46.3)	3875 (41.3)
Ga-adangbe	397 (8.8)	344 (7.1)	519 (46.8)
Ewe	718 (16.0)	646 (13.3)	1118 (11.9)
Guan	104 (2.3)	71 (1.5)	256 (2.7)
Mole-dagbani	492 (11.0)	510 (10.5)	2270 (24.2)
Other	394 (8.8)	1032 (21.3)	1356 (14.4)
**Religion**			
No religion	531 (11.8)	340 (7.0)	273 (2.9)
Catholic	765 (17.1)	775 (16.0)	1341 (14.3)
Other Christian	2381 (53.1)	2724 (56.3)	5828 (62.0)
Muslim	445 (9.9)	642 (13.3)	1726 (18.4)
Traditional	363 (8.1)	362 (7.5)	227 (2.4)
**Region**			
Western	392 (8.7)	519 (10.7)	1027 (10.9)
Central	463 (10.3)	447 (9,2)	941 (10.0)
Greater/Accra	598 (13.3)	692 (14.3)	999 (10.6)
Eastern	703 (15.8)	550 (11.4)	906 (9.6)
Volta	500 (11.2)	439 (9.1)	795 (8.5)
Ashanti	821 (18.3)	629 (13.0)	1040 (11.1)
B/A	500 (11.2)	309 (6.4)	1005 (10.7)
Northern	508 (11.3)	1258 (26.0)	2682 (28.6)
**Age at first marriage**			
Below 15	480 (13.3)	354 (9.4)	623 (9.8)
15-19	2242 (62.3)	2131 (56.8)	2960 (46.6)
20-24	735 (20.4)	964 (25.7)	1862 (29.3)
25+	141 (3.9)	302 (8.1)	910 (14.3)

Table 1 also shows an improvement in the overall level of education of the women. For example, the proportions of women with secondary education increased from 7% in 1988 to 52% in 2014, while the proportion of women with tertiary education increased from under 1% (0.9%) in 1988 to 5.5% in 2014. The proportion of married women decreased from 80.2% in 1988 to 67.4% in 2014. Table 1 also shows that in terms of ethnic identification, the most dominant group is the Akan ethnic group and the Guan followed by Ga/Adangbe who constitute the smallest and second smallest ethnic groups respectively in the three surveys.

As far as religion is concerned, other Christians (Protestants) are in the majority, followed by Catholics, while women who profess traditional religion and women with no religion constitute the lowest proportion. Women who reside in the Ashanti and Northern regions are the majority across the three survey periods. Women in Ashanti region were the highest in 1988 and Northern region in 1998 and 2014. Table 1 also reveals that most women married between the ages of 15-19 years in all the three surveys.

### Median age at first birth

**Figure 1.**
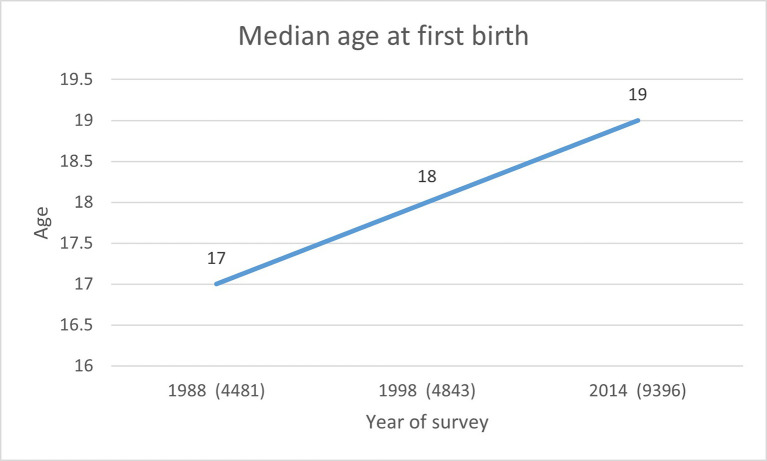
Median age at first birth among women aged 15-45 years in Ghana in 1988, 1998 and 2014.

Figure 1 shows the median age at first birth among women. The figure shows that the median age of the women is in a narrow range of 17 to 19 for the three survey years. Overall, the figure shows that trends in the median age at first birth increased by one year between 1988 and 1998 and by one year again between 1998 and 2014.

### Trends and differentials in median age at first birth

**Figure 2.**
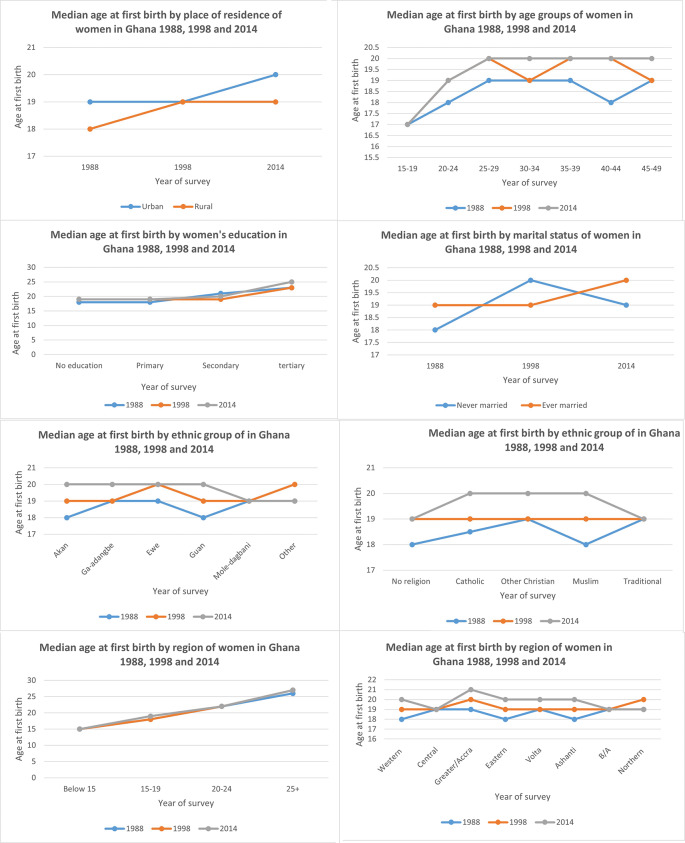
The median age at first birth by selected characteristics of women.

Figure 2 shows the trends and differentials in median age at first birth by the socio-demographic characteristics of the women. For women in the age group 40-44 years, there was a two-year increase in the age at first birth from 18 years in 1988 to 20 years in 2014. Figure 2 also shows that the median age at first birth was lowest (17 years) among women of the youngest (15-19) age cohorts for all the three surveys.

There is a positive relationship between the level of urbanization and the timing of first birth in Ghana. For example, there was a gradient increase in the median age at first birth from 19 years in urban areas in 1988 to 20 years in 2014. In rural areas, the median age at firth birth also increased by only one year over the survey period.

As far as the effect of education is concerned, Figure 2 shows that although the median age at first birth increased from 23 years in 1988 and 1998 among women with tertiary education to 25 in 2014, it remained relatively unchanged for the other educational groups. The differentials in age at first birth by level of education show that age at first birth increased with the level of education except for women with secondary education for whom the mean age dropped from 21 in 1988 to 19 in 1998, and then to 20 years in 2014. Women with no education and primary education had the lowest age at first birth, while women with tertiary education continue to have the highest age at first birth for all the survey years.

As far as marital status is concerned, Figure 2 shows that the median age at first birth for ever-married women increased marginally by 1 year for each survey year. There was an increase in age at first birth for ever-married women from 19 in 1998 to 20 in 2014. The age at first birth also increased among all ethnic groups between 1988 and 2014. However, as shown in Figure 2, the Ewe and Ga/Adangbe ethnic groups delayed the longest in transitioning to parenthood, while the Mole/Dagbani ethnic group reported the lowest increase in age at first birth over the survey period.

In terms of religion, there was a median increase of two years in age at first birth for all religious groups from 19 years in 1988 to 21 years in 2014, except for women with no religion and women in other religious groups. Specifically, for each survey year, Catholics, other Christians and Muslims experienced higher and similar increases in age at first birth from 19 years in 1988 to 20 years in 1998 and 21 years in 2014.

Generally, there is an increase in the median age at first birth for all regions except for the “Northern regions” where the median age at first birth increased from 19 years in 1988 to 20 years in 1998. In the northern regions, there was a decrease to 19 years in 2014, while the Volta region had the highest increase in the median age at first birth from 19 years in 1988 to 20 years in 1998 and remained at 20 years in 2014.

The age at which a woman marries affects the timing of the onset of parenthood in Ghana. Figure 2 shows that as a woman postpones the age at first entry into a marital union, there is a tendency to postpone the onset of parenthood. For example, the median age at first birth for women who married before 15 years of age in 1988 is 15 years compared to a median age at first birth for women who married at above 25 years and above. Moreover, in 2014, the median age at first birth for women who married at above 25 years increased from a median age of 26 years in 1988 to a median age of 27 years in 1998 and 2014.

### The multivariate analysis

The results of the multivariate analysis of the risk and protective factors of the timing of birth for the three waves of the data in Ghana are presented in Table 2. The table shows that almost all the selected social and demographic factors (age, education, ethnicity, region and age at first marriage) were significantly associated with age at first birth across the three surveys. However, the only factor that had a strong, consistent impact on the timing of first birth in Ghana is the age of a woman. As the table shows, the hazard risk of early childbirth consistently decreased as age increased in all the years of the surveys except in the case of age group 40-44 in 1988, 1998 and 2014. For instance, the hazard risk of early childbirth was 0.47, 0.46, and 0.47 times lower among women between the ages of 40-44 years compared to women between the ages of 15-19 years in 1988, 1998 and 2014 respectively.

**Table 2.** Cox proportional hazard regression of age at first birth among selected socio-demographic variables in Ghana using 1988, 1998 and 2014 Ghana DHS.

	1988		1998		2014	
	Hazard ration	Confidence interval	Hazard ration	Confidence interval	Hazard ration	Confidence interval
**Variables**						
**Age**						
15-19	1		1		1	
20-24	0.58	0.47-0.70 *	0.58	0.46-0.73 *	061	0.49-0.77 *
25-29	0.48	0.40-0.58 *	0.50	0.40-0.62 *	0.47	0.38-0.59 *
30-34	0.50	0.41-0.61 *	0.49	0.39-0.62 *	0.48	0.38-0.60 *
35-39	0.45	0.39-0.55 *	0.44	0.35-0.55 *	0.42	0.33-0.52 *
40-44	0.47	0.38-0.58 *	0.46	0.37-0.58 *	0.47	0.37-0.57 *
45-49	0.38	0.31-0.47 *	0.44	0.35-0.56 *	0.41	0.33-0.52 *
**Place of residence**						
Urban	1		1		1	
Rural	1.04	0.96-1.13	0.98	0.90-1.07	1.18	0.89-1.25
**Level of education**						
No education	1		1		1	
Primary	0.92	0.84-1.00	0.98	0.88-1.09	0.97	0.89-1.05
Secondary	0.67	0.57-0.80 *	0.89	0.81-0.98 *	0.77	0.71-0.83 *
Tertiary	0.77	0.52-1.15	0.87	0.68-1.12	0.53	0.45-0.61 *
**Ethnic groups**						
Akan	1		1		1	
Ga-adangbe	1.00	0.86-1.16	0.83	0.71-0.97 *	0.98	0.88-1.09
Ewe	0.97	0.84-1.11	0.96	0.84-1.09	0.96	0.87-1.07
Guan	0.66	0.52-0.88 *	1.08	0.81-1.43	0.91	0.76-1.08
Mole-dagbani	0.75	0.63-0.86 *	0.91	0.74-1.11	0.82	0.73-0.92 *
Other	0.76	0.64-0.90 *	0.92	0.79-1.08	0.91	0.81-1.01
**Religion**						
No religion	1		1		1	
Catholic	1.08	0.95-1.23	1.01	0.87-1.18	0.89	0.75-1.04
Other Christian	1.02	0.91-1.15	0.97	0.84-1.11	0.94	0.81-1.08
Muslim	1.05	0.90-1.22	0.95	0.80-1.13	0.89	0.76-1.05
Traditional	0.90	0.77-1.04	0.99	0.81-1.21	0.89	0.72-1.09
**Region**						
Western	1		1		1	
Central	1.08	0.93-1.26	0.99	0.86-1.14	1.10	0.99-1.23
Greater/Accra	0.99	0.83-1.18	0.86	0.73-1.01	0.99	0.89-111
Eastern	1.22	1.05-1.42 *	0.96	0.83-1.14	1.13	1.00-1.26 *
Volta	1.06	0.88-1.28	0.96	0.80-1.14	0.94	0.82-1.09
Ashanti	0.94	0.81-1.08	0.95	0.83-1.09	1.05	0.95-1.16
B/A	1.02	0.88-1.19	1.03	0.88-1.21	1.16	1.03-1.31 *
Northern	1.12	0.92-1.36	0.92	0.79-1.08	1.10	0.97-1.25
**Age at first marriage**						
Below 15	1		1		1	
15-19	0.33	0.30-0.37	0.32	0.29-0.37 *	0.32	0.29-0.35
20-24	0.11	0.10-0.13	0.11	0.10-0.13 *	0.12	0.11-0.13
25+	0.05	0.44-0.07	0.05	0.04-0.06 *	0.06	0.05-0.64

* Significant results.

The effect of education on the onset of parenthood was not always linear as the theory of modernization would predict, even though on the whole, educational attainment appeared to exert a negative influence on age at first birth. The inconsistent effect of education on age at first birth is shown by the fact that in 1988 there was no significant difference between women with education and those with primary education and tertiary level of education, on the other hand. Moreover, in 1988, women with secondary education were more likely to delay parenthood compared to their counterparts with tertiary education and those without education which is the reference category.

But, as the table clearly shows, over time, the effect of education on the timing of parenthood is unmasked as evidenced by the fact that women with primary, secondary and tertiary education were all more likely to initiate parenthood later than those without education. For example, women secondary education had 0.67, 0.89- and 0.77-times lower hazard risk of early birth than women without any formal education in 1988, 1998, and 2014 respectively. Furthermore, women with tertiary education had 0.77 times lower hazard risk of early childbearing compared to women without education in 1998 compared to women with no education.

Cultural values and norms largely shape the attitudes and behaviors of individual members of ethnic groups towards social institutions like the family in terms of their structure and process. As Table 2 shows, ethnicity affects the timing of the onset of parenthood in Ghana in significant ways. With the exception of the Ewe ethnic group which is not significantly different from the Akan ethnic group (the reference category), all the remaining ethnic groups, initiated parenthood later than the Akan ethnic group, but the statistical significance varied across the three surveys. For instance, women from the Ga-Adangbe ethnic group had 0.83 times lower hazard risk of early childbirth compared to women from the Akan ethnic group in 1998. This finding is similar to that of women of the Guan ethnic group who had 0.66 lower times the hazard risk ratio of initiating parenthood early than their counterparts from the Akan ethnic group in 1988. Moreover, women from the Mole-Dagbani ethnic group had 0.75 and 0.82 lower hazard risk of early birth compared to their counterparts from the Akan ethnic groups in 1988 and 2014 respectively.

While as a geographic entity region largely reflects local social, economic, and demographic conditions, it often has an independent effect on various social phenomena as shown clearly by the findings in Table 2 although this effect is not consistent over time. For example, even though women in the Eastern and Brong/Ahafo regions all had higher hazard risks of early birth regimes compared to women in the Western region, these effects are not consistent over the three surveys. While women in the Eastern region started birth early in 1988 and 2014 (HR; 1.22 and 1.13) respectively, this tendency is observed for women in the Brong/Ahafo region (HR; 1.16) only in 2014.

While age at first marriage may reflect changing social and economic conditions in the society, Table 2 shows that after factoring in such socio-economic factors as education and residence, the age at which a woman first married had the most impact on the age at which she had her first child as shown by the findings for the 1998 survey. For example, women who married at 15-19, 20-24, and above 25 years had lower hazard risk of 0.32, 0.11, and 0.05 respectively to times to have early childbirth compared to women who married before 15 years.

## Discussion

The present study sought to examine the effect of selected social and demographic factors on the timing of the onset of parenthood among women in Ghana using three waves of the Demographic and Health Survey data. Specifically, we employed the Cox proportional Hazard Model to examine the effects of place of residence, educational attainment, ethnic background, region of residence, religious affiliation, and age at first marriage, and age at first birth of women aged 15 to 49 years old. The results of this study have shown that socio-demographic factors (especially age, education, ethnicity, region and age at first marriage) are important in predicting age at first birth.

Our results are consistent with the life course theory. The life course theory provides a useful means of illuminating changes in age at first birth in Ghana over time. This theoretical perspective allows the link between social context and age at which a woman first gives birth. This link occurs in the interactions of women in contexts in which she lives such as schools, family, employment, and religion in which she lives. We found that the transitions into motherhood varied among the ethnic groups and regions of residence. A possible explanation of this variation could be the traditional norms and values that govern the formation of family or childbearing.

Our finding regarding the association of region and age at first birth is similar to the findings of Gurmu and Etana (2014) conducted in Ethiopia and with the outcomes of Fagbamigbe and Idemudia (2016) in a study conducted on survival analysis and prognostic factors of timing of first childbirth among women in Nigeria.

The finding that women between the ages of 15-19 years have the highest risk of early childbirth compared to all other age groups is consistent with the findings of a Nigerian study which reported that majority of women in the study have had their first birth before the age of 20 years ( Fagbamigbe and Idemudia 2016), and another study in Bangladesh which observed that 72.8% of the women in the study had their first child before the age of 20 years ( Aminu Haque and Sayem 2009). It is possible that younger women may not have the power to negotiate safe sex practices and they may not be able to partake in fertility decision making in a case where they marry at a younger age.

Early marriage is a strong predictor of age at first birth. Consistent with a previous study in Ghana on Statistical distribution and determinants of mother’s age at first birth ( Logubayom and Luguterah 2015), this finding that the earlier a woman is first married, the earlier she bears her first baby. This is because early marriage increases the risk of early sexual initiation that is likely to increase the risk of pregnancy. This is in line with our results that show that marrying before the age of 15 years increases the risk of early age at first birth compared to marrying after the age of 15 years. This finding is also consistent with the findings of Mohammad Manjur Alam (2015) on marriage to first birth interval and its associated factors in Bangladesh.

Our results suggest that having higher education is consequential for women’s age at first birth. Higher level of education delays the timing of age at first birth. We found that women with no former education have higher risk of early childbirth, a finding which is consistent with the findings in Ghana (see e.g., Logubayom and Luguterah 2015), Ethiopia ( Gurmu and Etana 2014), and Brazil ( Marteleto and Dondero 2013). The negative association between education and age at first birth is possibly because education is a competing activity to childbirth. In addition, education empowers women in terms of arming them with knowledge about the importance of contraception and a greater say in fertility decisions in the household.

The present study has provided empirical evidence on the age at first birth in Ghana. Despite the findings and the contribution of this study to reproductive health, some important limitations should be considered while interpreting the findings of this study. The cross-sectional form of this study, which makes it challenging to infer causation with some of the variables under investigation, is a glaring weakness. The socio desirability of the surveys is another restriction that should be taken into account. Social desirability may have reduced reporting or led to inaccurate responses on the questionnaire about age at first birth.

## Conclusion

In conclusion, the present study has shown that in tandem with broader transformation of the Ghanaian society as a result of such modernizing forces as education, urbanization and their concomitant attitudinal and demographic changes, the timing of first childbirth is changing in the direction of a late childbirth regime. This change in the age at which a woman has her first child is bound to affect the structure of the Ghanaian family through its effect on fertility levels.

## Data Availability

Data used in this study are from the cross-sectional individual women dataset of the Ghana 1988, 1998, and 2014 demographic and health surveys, available from the Demographic and Health Survey (DHS) website https://dhsprogram.com/. Access to the dataset requires registration and is granted only for legitimate research purposes. A guide for how to apply for dataset access is available at: https://dhsprogram.com/data/Access-Instructions.cfm.
