# Supplementary material for: Socio-demographic correlates and trends in the timing of the onset of parenthood among women of reproductive age in Ghana: evidence from three waves of the demographic and health surveys
Source: F1000Res. 2023 Feb 10;12:157. [Version 1] doi: 10.12688/f1000research.130349.1 (PMC10390794; doi:10.12688/f1000research.130349.1)
Supplement: Peer review history

# Peer review: Reviewer response for version 1

**DOI:** 10.5256/f1000research.143099.r181169

**Published:** 2023-07-31

**Authors:** Babatunde Gbadebo

**Affiliations:** 1 Department of Epidemiology and Medical Statistics, University of Ibadan, Ibadan, Oyo, Nigeria

**General comment:** 

 There are a few typographical and editorial errors that should be carried out. For instance, towards the end of the introduction, a statement reads: ’Other reported effects of early age at first birth are truncated education which has ramifications for the socioeconomic wellbeing of women and their children’ instead of ‘Other reported effect of early age at first birth is truncated education which has ramifications for the socioeconomic wellbeing of women and their children’.

 Similarly, the word “socio-demographic” is included in the title of the article but there were occasions that the authors wrote sociodemographic instead of socio-demographic; there is need for consistency.

 In the sixth paragraph of the discussion, 'former education' should be written as formal education.

**Abstract**: Results - kindly specify the age of the women you were reporting. Is it the age of respondents or the median age at the onset of birth/parenthood?

**Keywords: **Keywords should be between 3 and 5: you can delete sociodemographic, Demographic and Health Surveys, and Cox proportional.

**Introduction**: Don't start the second paragraph with 'because'.

**Objective of the study**: Kindly delete lines 1 to 4a; it is more or less part of the study's justification, rather than the objective of the study. 

**Research tools and data collection**: Kindly specify clearly which of the questionnaires you used for your study - or did you use all the questionnaires for your study?

**Statistical analysis**: Specify the univariate analysis tool used.

**Results: **Don't start your report with 'as far as'; kindly recast those statements that start with 'as far as'.

**Discussion**: Kindly recast paragraph 5 of your discussion. The discussion should start from the general principle (if there is a need), your findings, how it relates to previous findings, and the implication of your findings

**References**: Kindly check your fourth reference. Reference should be written as: surname first, followed by the initial(s).

Is the work clearly and accurately presented and does it cite the current literature?

Yes

If applicable, is the statistical analysis and its interpretation appropriate?

Yes

Are all the source data underlying the results available to ensure full reproducibility?

Yes

Is the study design appropriate and is the work technically sound?

Yes

Are the conclusions drawn adequately supported by the results?

Yes

Are sufficient details of methods and analysis provided to allow replication by others?

Yes

Reviewer Expertise:

Reproductive health, Child mortality, Applied Demography, Female genital cutting

I confirm that I have read this submission and believe that I have an appropriate level of expertise to confirm that it is of an acceptable scientific standard.

# Peer review: Reviewer response for version 1

**DOI:** 10.5256/f1000research.143099.r185522

**Published:** 2023-07-25

**Authors:** Christine Chawhanda

**Affiliations:** 1 University of Zimbabwe, Harare, Harare Province, Zimbabwe

The references cited in the literature are from the 1990s to 2016. Perhaps the authors could include most recent references (2019, 2020, 2021, 2022)?

 Under results on continuation of figure 2 titled median age at first birth by region, the last figure to the left the regions are not shown in the figure (the figure does not show age at first birth by region), however, the figure after this (to the right) is the one that shows age at first birth by region.

 The multivariate results on region, kindly revisit the results that you have marked as significant.

 Under discussion, The Life course theory allows for analysing people's lives within structural, social, and cultural contexts. In your discussion, perhaps you could acknowledge the importance or the role of culture (societal expectations) in the timing of pregnancy. Once married, the society may expect the woman to get pregnant and discourage family planning soon after marriage.

 Based on your findings and discussion, what recommendations can you provide for Ghana to consider.

Is the work clearly and accurately presented and does it cite the current literature?

Partly

If applicable, is the statistical analysis and its interpretation appropriate?

Yes

Are all the source data underlying the results available to ensure full reproducibility?

Yes

Is the study design appropriate and is the work technically sound?

Yes

Are the conclusions drawn adequately supported by the results?

Partly

Are sufficient details of methods and analysis provided to allow replication by others?

Yes

Reviewer Expertise:

Sexual and reproductive health, fertility, teenage pregnancy, migration, public health policy and systems

I confirm that I have read this submission and believe that I have an appropriate level of expertise to confirm that it is of an acceptable scientific standard, however I have significant reservations, as outlined above.

# Peer review: Reviewer response for version 1

**DOI:** 10.5256/f1000research.143099.r185520

**Published:** 2023-07-20

**Authors:** Adriana A E Biney

**Affiliations:** 1 Regional Institute for Population Studies, University of Ghana, Legon, Ghana

This is a well written manuscript that assesses the sociodemographic correlates and trends in the timing of the onset of childbearing among women in Ghana. I have listed below a few comments for the authors that I believe will help improve the manuscript.

**Abstract**

 The sample size(s) should be included in the abstract as this provides useful information for those simply reviewing abstracts.

**Introduction**

 In the second paragraph, line 12, the sentence begins with the word ‘Although’ however as you read on this does not fit and should be revised.

 Another subsection under the Introduction should be included providing some contextual background on childbearing in Ghana, so readers can understand the Ghanaian context regarding fertility. 

 In their discussion of the problem warranting the study, the authors do not clearly state why it is important to assess trends in onset of childbearing time, and why it is important to assess the correlates of age at first birth. We understand that the age at first birth comes with certain consequences and fertility implications but clear statements as to why it is important to know the socio-demographic trends in the age at first birth of women are needed. Perhaps it could be that this is not an important thing to know hence studies on this being non-existent.

 As the manuscript reports on what has been done and not done on the topic, the Introduction could also include some other references that discuss the topic of transitioning to adulthood and the onset of childbearing. For example, Beguy *et al.* (2011) ^1^. Most of the papers cited are quite old, those for Ghana, especially, which was even mentioned by the authors. They should attempt to include a few more current papers.

**Theoretical framework**

 At the end of the discussion on the theory and the various factors the literature has indicated as being linked to age at first birth, it could be helpful to end the section with a brief paragraph summarizing the key variables based on the review of the literature. Readers would then understand your justification for including these variables in the study. The authors could again tie this back to the life course theory.

 In paragraph 4 – the authors refer to the Ghana Statistical Service (2009). I suggest they mention to the DHS report or just report the information and provide the citation after (GSS, GHS and ICF Macro, 2009). I also wonder why not all the GDHS reports for which their data were analysed were included in the references but only the 2008 GDHS was cited.

**Methods**

 The authors could include a statement that they solely used the women’s file for their analysis. This is so that readers are not confused as all the questionnaires – women’s, men’s and household were initially mentioned.

 As the authors describe their sociodemographic variables, they report marriage and refer to it as a dichotomous variable of marital status, however, later on the variable on age at first marriage is used for the bivariate graphs and multivariate analysis. Age at first marriage is also not described along with the other measures.

 The authors should write B/A in full and capitalize the first letters for the Western, Central, Greater Accra, and Northern regions.

 The readers should report in their Statistical Analysis section how they conducted the analysis to report the trends they presented in graphs. This was not explained.

 Readers may benefit from a sentence under the Statistical Analysis section explaining whether the assumptions for use of the Cox proportional hazard model were met.

**Results**

 The authors should clarify the samples used for the different analyses conducted. More specifically, in order to compute the median ages at first birth across the three years, were all women – even women with no births – included? I understand they were included in the regression analysis as this variable was dichotomized to include those with and without births, but I do not understand what was done to obtain the median ages for the different variables if these women do not have an age at first birth.

 Other Christians are not all Protestants, as some are Latter Day Saints and Jehovah’s Witnesses and are not historically from the same line of Christianity.

 For the various graphs indicating trends in age at first birth by sociodemographic variables, the data values could be indicated. Also, some graphs indicate the Year of Survey as the x-axis when it is not. This occurs in figure 2 for age group of women (on page 8), and all graphs in Figure 2 continued (on page 9). Also, age at first marriage graph has been titled wrongly indicating region instead.

 The authors need to explain the rationale for the different line graphs in Figure 2, so that readers can better understand the results being displayed on the graphs. Some graphs do not indicate the trends across the three DHS waves but rather changes in the categories by the year of survey. Explanations are given but the authors should have another look especially at the graphs that display nominal data (religion, ethnic group, and region graphs) but present these in a trend format. Perhaps bar graphs would be best to represent these data instead of the line graph.

 On Table 2 – the authors use the term hazard ration instead of ratio.

 The final paragraph under the multivariate results explaining the age at first marriage variable reports that after age and education were factored in, it had an impact on age at first marriage. This statement indicates that some sort of stepwise analysis was done as variables were included in the model. If so, this must be stated.

 I wonder about some control variables being included in the model. I understand the authors were only considering important sociodemographic variables which were in line with their theoretical framework as the main correlates. However, a variable such as wealth quintile could be included as a control and not a correlate in the study.

 The final paragraph in the Results section reports age at first marriage findings. On line 4 in that paragraph, the sentence says “…had a lower hazard of 0.32, 0.11, and 0.05 respectively to times to have…”. This should be revised.

**Discussion**

 The results on findings with the age at first marriage variable indicate only 1998 results were significant. Can the authors posit as to why the 1988 and 2014 results were not also significant despite indicating very similar ratios.

 Final conclusions about the trends need to be included since this was a major aim of the study. What can the authors tell readers about the changes over time concerning age at first birth and the different correlates.

 Are there any recommendations the authors can suggest? The results on education and early marriage may be important for policymakers and programmers.

 The first reference to socio desirability should also be social desirability.

Is the work clearly and accurately presented and does it cite the current literature?

Partly

If applicable, is the statistical analysis and its interpretation appropriate?

Partly

Are all the source data underlying the results available to ensure full reproducibility?

Yes

Is the study design appropriate and is the work technically sound?

Yes

Are the conclusions drawn adequately supported by the results?

Partly

Are sufficient details of methods and analysis provided to allow replication by others?

Yes

Reviewer Expertise:

Sexual and reproductive health, health systems

I confirm that I have read this submission and believe that I have an appropriate level of expertise to confirm that it is of an acceptable scientific standard, however I have significant reservations, as outlined above.

## References

[1] : Timing and Sequencing of Events Marking the Transition to Adulthood in Two Informal Settlements in Nairobi, Kenya. *Journal of Urban Health* .2011;88(S2) : 10.1007/s11524-011-9547-8 318-340 10.1007/s11524-011-9547-8 PMC313224021340662
